# IL-6 Enhances the Activation of PI3K-AKT/mTOR-GSK-3β by Upregulating GRPR in Hippocampal Neurons of Autistic Mice

**DOI:** 10.1007/s11481-024-10111-3

**Published:** 2024-03-27

**Authors:** Heli Li, Xinyuan Wang, Cong Hu, Jinru Cui, Hao Li, Xiaoping Luo, Yan Hao

**Affiliations:** 1 Division of Child Healthcare, Department of Pediatrics, Tongji Hospital, Tongji Medical College, Huazhong University of Science and Technology, Wuhan, 430030 China; 2 Tongji Medical College, Huazhong University of Science and Technology, Wuhan, 430030 China; 3 Department of Pediatrics, Tongji Hospital, Tongji Medical College, Huazhong University of Science and Technology, Wuhan, 430030 China

**Keywords:** Autism, IL-6, GRPR, Hippocampus, PI3K

## Abstract

Autism spectrum disorder (ASD) is a neurological disorder associated with brain inflammation. The underlying mechanisms could be attributed to the activation of PI3K signaling in the inflamed brain of ASD. Multiple studies highlight the role of GRPR in regulating ASD like abnormal behavior and enhancing the PI3K signaling. However, the molecular mechanism by which GRPR regulates PI3K signaling in neurons of individuals with ASD is still unclear. In this study, we utilized a maternal immune activation model to investigate the effects of GRPR on PI3K signaling in the inflamed brain of ASD mice. We used HT22 cells with and without GRPR to examine the impact of GRP-GRPR on the PI3K-AKT pathway with IL-6 treatment. We analyzed a dataset of hippocampus samples from ASD mice to identify hub genes. Our results demonstrated increased expression of IL-6, GRPR, and PI3K-AKT signaling in the hippocampus of ASD mice. Additionally, we observed increased GRPR expression and PI3K-AKT/mTOR activation in HT22 cells after IL-6 treatment, but decreased expression in HT22 cells with GRPR knockdown. NetworkAnalyst identified GSK-3β as the most crucial gene in the PI3K-AKT/mTOR pathway in the hippocampus of ASD. Furthermore, we found that IL-6 upregulated the expression of GSK-3β in HT22 cells by upregulating GRP-GRPR. Our findings suggest that IL-6 can enhance the activation of PI3K-AKT/mTOR-GSK-3β in hippocampal neurons of ASD mice by upregulating GRPR.

**Graphical Abstract:**

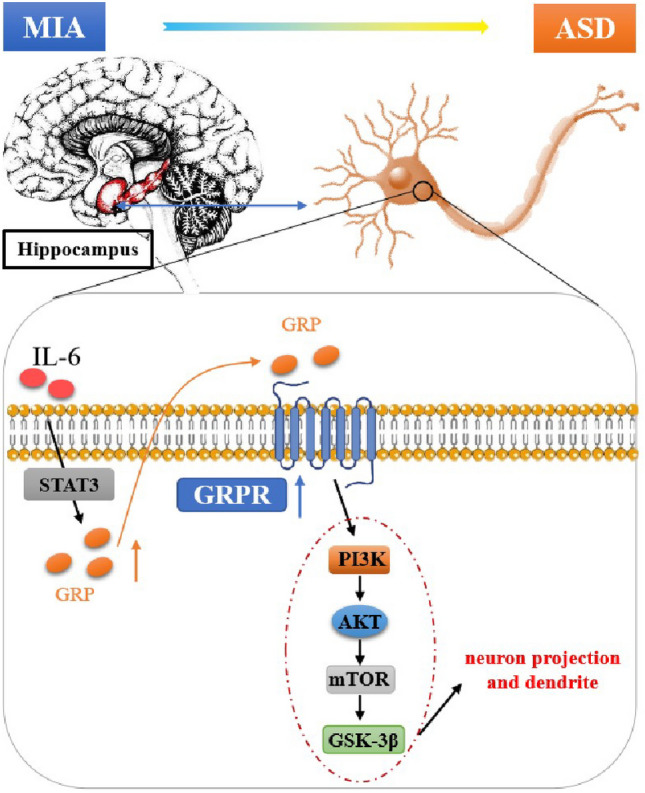

## Introduction

Autism Spectrum Disorder (ASD) is a group of neurodevelopmental disorders characterized by impaired social communication, restrictive interests and repetitive behaviors (Battle 2013). The prevalence of ASD in 8-year-old children is approximately 1 in 36, as reported by the American Centers for Disease Control and Prevention (CDC) in 2021 (Maenner et al. 2023), this rate is rapidly increasing. Large epidemiologic studies have shown that maternal immune activation (MIA) caused by prenatal exposure to bacterial or viral infections increases the risk of ASD (Careaga et al. 2017; Lombardo et al. 2018). Animal researches have revealed that MIA exacerbates ASD-like behavioral abnormalities by causing brain inflammation in offspring (Cieślik et al. 2023), and they have also identified some potential mechanisms, such as the inflammatory injury of cytokines like interleukin (IL)-6 (Brown et al. 2014; Lesh et al. 2023) and IL-17 (Bagcioglu et al. 2023; Sarieva et al. 2023) in the brain. It is important to note that blocking IL-6 (Smith et al. 2007) or IL-17 A (Reed et al. 2020) signaling effectively alleviated behavioral abnormalities in ASD mice, although the specific neural mechanisms are still unclear. Therefore, gaining a deeper understanding of the mechanisms by which inflammatory factors affect neurons in ASD is crucial for developing accurate diagnosis and effective interventions for ASD.

Increasing investigations have demonstrated that phosphatidylinositol 3 (PI3K) signaling is an essential intracellular signaling system activated by brain inflammation (Le Belle et al. 2014; Hodges et al. 2021). PI3K is a family of lipid kinases that phosphorylate the 3′-hydroxyl group of phosphatidylinositide and phosphoinositides, controlling the activation of protein kinase B (PKB, also named AKT) and the mammalian target of rapamycin (mTOR) (Bilanges et al. 2019). The PI3K-AKT/mTOR signaling pathway plays a role in synaptogenesis, corticogenesis, and other neuronal cerebral processes (Gilbert and Man 2017), and its dysregulation has been implicated in the progression of ASD (Chen et al. 2014; Wang et al. 2022). Abnormal activation of the PI3K-AKT/mTOR signaling pathway in hippocampal neurons has been shown to result in repetitive behavior, social behavior deficits, and serotonin impairment (Lugo et al. 2014). In a study with ASD rats, inhibition of the PI3K-AKT/mTOR signal completely reversed social defects and repetitive behavior (Xing et al. 2019). Dysregulation of PI3K-AKT has also been observed in a human induced pluripotent stem cell-derived ASD neuron model, and the degree of dysregulation is related to the severity of ASD symptoms in young children (Gazestani et al. 2019). Therefore, investigating the factors that can regulate the activation of the PI3K-AKT/mTOR signaling pathway in specific brain regions will provide insights into the pathogenesis of ASD.

Gastrin-releasing peptide (GRP) receptor (GRPR), a member of the G protein coupled receptor (GPCR), regulates the activation of the PI3K-AKT pathway in neurons (Pereira et al. 2015; Sun et al. 2023). GRPR is expressed in neurons in multiple brain regions, controlling the circadian cycle, anxiety, fear, stress, and modulation of memory (Roesler et al. 2006a, b). Specifically, GRPR is present in hippocampal neurons and plays a role in regulating synaptic transmission, which can contribute to cognitive damage associated with the hippocampal area (Yang et al. 2017). In a study on rats, neonatal GRPR blockade resulted in reduced sociability, restrictive interests, motor stereotypies, and an enhanced learned fear response (Merali et al. 2014). Furthermore, GRPR signaling, known for its involvement in itch transmission (Yu et al. 2017), has been found to increase in neurons following stimulation of inflammatory factors (Liu et al. 2023). It is worth noting that MIA caused by Poly-ic in mice led to an increase in proinflammatory cytokines in the fetal brain, particularly IL-6, resulting in autism-like behavioral symptoms in offspring (Jones et al. 2017; Horváth et al. 2019). Therefore, we hypothesize that GRPR could be upregulated in ASD mice exposed to MIA and exacerbate autism-like behavior by activating the PI3K-AKT/mTOR signaling pathway.

In this study, we utilized maternal immune activation to create a mouse model of ASD. Our objective was to investigate any changes in GRPR expression and the activation of the PI3K-AKT/mTOR signaling pathway in the brain. Additionally, we conducted in vitro experiments to suppress the expression of GRPR in mouse hippocampal neuron HT22 cells, in order to examine the role of GRPR in the activation of the PI3K-AKT/mTOR signaling pathway. By elucidating a potential mechanism through which GRPR can influence neurons in ASD, our study may contribute to identifying a promising therapeutic target for ASD treatment.

## Methods

### Mice

C57BL/6 mice were obtained from HFK Bioscience (Beijing, China) and housed in a specific pathogen-free environment. Timed pregnancies were established by pairing a male and female overnight, and the mid-day of the next morning was designated as embryonic day 0.5 (E0.5). Maternal immune activation (MIA) was performed according to a previously described method (Li et al. 2022). Briefly, mice were mated overnight, and the presence of seminal plugs was checked every morning, which was recorded as embryonic Day 0.5 (E0.5). On E12.5, pregnant mice received an intraperitoneal injection of 20 mg/kg of Poly-ic (#P9582, Sigma). Only male mice were used in all studies involving adult offspring, and their behavior was assessed at 6 weeks of age.

### Behavioral Testing

#### Three-chamber Social Interaction Test

Social preference was assessed using a 3-chamber Plexiglas arena (40 × 60 cm). The arena was divided into 3 equal chambers, each measuring 20 × 40 cm, with a 4 × 4 cm square opening allowing the test mice to move between chambers. The experiment consisted of three phases. In Phase 1, the test mouse had free access to the entire arena for habituation. In Phase 2, the test mouse was briefly placed in the center chamber while an unfamiliar stranger mouse was placed in one of the cages. The test mouse was given 10 min to explore the arena. In Phase 3, the cage containing the unfamiliar stranger mouse was moved to another chamber, and the cage that previously held the stranger mouse in Phase 2 was now occupied by a novel mouse. The Ethovision XT 10 system (Noldus) was used, connected to an overhead camera, to track and record the behavior of the test mouse. The discrimination index in Phase 2 was calculated as the difference between the time spent exploring the stranger mouse and the time spent exploring an empty cage, divided by the total time spent exploring social stimuli. Similarly, the discrimination index in Phase 3 was calculated as the difference between the time spent exploring the novel mouse and the time spent exploring a familiar mouse, divided by the total time spent exploring social stimuli.

#### Open Field

In the open-field apparatus (43.2 cm × 43.2 cm), mice were placed in a corner and allowed to move freely. Data were collected using the MED Associates’ Activity Monitor Data Analysis software on a PC. Prior to testing, the mice were not exposed to the chamber. Individual data were recorded for each animal over a 5-minute period.

#### Marble Burying Test

The marble burying test was designed based on the method described by Malkova et al. (2012). Clean cages (36.7 × 14.0 × 20.7 cm) were filled with 5 cm corn cob bedding. Then, 20 blue glass marbles were gently placed on the surface of the bedding in a 4 × 5 arrangement, evenly spaced from each other. The testing animals were placed in the area, and the number of marbles buried within a 30-minute testing period was measured. Marbles were considered buried if they were covered by at least 60% of the bedding.

#### Elevated Plus Maze

The plus maze consisted of two walled arms (the closed arms, 35 cm L × 6 cm W × 22 cm H) and two open arms (35 cm L × 6 cm W). Mice were placed in the center section and allowed to freely explore the maze. Their activity was monitored using ImageEP software66. The time spent in the open versus closed arms during the 5-minute period was recorded.

### Western Blot

Hippocampus isolated from the mouse brains were homogenized in ice-cold lysis buffer containing 50 mM Tris-HCl, 150 mM NaCl, 1% NP-40, 2 mM EDTA, 1 mM Na-orthovanadate, (pH 7.4), and proteinase inhibitor mixture (Thermo SCIENTIFIC, 1 mL/10 g tissue) and collected the homogenate to centrifuge at 1,000 g at 4 °C for 10 min. The following antibodies were used: anti-GRPR (ab188821), anti-PIK3R3 (ab238509), anti-AKT (4685 S), anti-p-AKT (Ser473) (12,694 S), anti-mTOR (2983 S), anti-β-Actin (66009-1-Ig), goat anti-rabbit labeled with HRP (ab205718), goat Anti-mouse labeled with HRP (ab205719). The Image-Pro plus software, version 6.0, from Media Cybernetics (Rockville, MD, USA) were used to determine the chemiluminescent and relative protein expression, respectively, which was represented as the density ratio vs. Actin.

### Immunofluorescence

After fixation with 4% PFA in PBS, the brain slice or HT22 cells were incubated with anti-GRPR, anti-p-AKT (Ser473) overnight. The cells were then washed with PBS and incubated with Alexa 488 or Alexa 596 conjugated secondary antibodies (Invitrogen) for 90 min. To label the nucleus, cells were also incubated with DAPI (Invitrogen) for 5 min. A laser-scanning confocal microscope (LSM 800; Zeiss) was used for fluorescence imaging.

### Microarray Source

Gene expression data from microarray studies of ASD were downloaded from the Gene Expression Omnibus (GEO) (https://www.ncbi.nlm.nih.gov/geo/). GSE178403 from GPL24247 included mRNA profile of 3 different brain regions (anterior cingulate cortex, dorsal hippocampus, ventral hippocampus) from 12 offspring of MIA mice treated by Poly-ic (Guma et al. 2021). The dataset was pre-processed using the log2 transformation and quantile normalization by the R package.

The enrichment network of differentially expressed genes (DEGs) and Over-Representation Analysis (ORA)-generated heatmaps were performed using Network Analyst (Zhou et al. 2019), a website that can generate tissue-specific Protein-Protein Interaction Networks (PPI) and gene co-expression networks. The network-based bioinformatics analysis utilized the Fisher’s method with a significance level of *p* < 0.05, as implemented in NetworkAnalyst, following the pipeline described by Li et al. (2022).

### RNA Quantification

The RNeasy^®^ Mini Kit (Qiagen^®^, Venlo, Netherlands) was performed to extract total RNA from collected tissues or cultured cells, which was then reverse transcribed into cDNA using the M-MLV reverse transcriptase (Invitrogen). Quantitative real-time polymerase chain reaction (qRT-PCR) analyses were carried out using a SYBR Green Real-time PCR kit (Toyobo, Osaka, Japan) in a LightCycler^®^ (Bio-Rad Laboratories, Hercules, CA, USA). Data were normalized to GAPDH, and fold changes were analyzed using the formula: 2^−Δ Δ Ct^.

Primer pairs used were as follows: IL-6: 5’-TACCACTTCACAAGTCGGAGGC-3’ and 5’-CTGCAAGTGCATCATCGTTGTTC-3’; GRPR: 5’-GTGGACCCTTTCCTGTCCTG-3’ and 5’-GGACTTGACCGTGCAGAAGA-3’; GRP: 5’-GAGCTCTCGCTCTTGCTGTT-3’ and 5’-GAGCTCTCGCTCTTGCTGTT-3’; GSK-3β: 5’-GAGAACCACCTCCTTTGCGG-3’ and 5’- TGGTTACCTTGCTGCCATCT-3’; GAPDH: 5’-TCTCCACACCTATGGTGCAA-3’ and 5’- CAAGAAACAGGGGAGCTGAG-3’.

### HT22 Knockout Cell Line

To establish GRPR knockout HT22 cells, specific CRISPR sgRNA for knock out of the GRPR gene was designed using the CRISPR Design web site (Feng Zhang laboratory, MIT, Cambridge, Massachusetts, USA; http://crispr.mit.edu). sgRNA was then cloned into the pSpCas9(BB)-2 A-GFP (#48138, Addgene, Cambridge, MA) (Ran et al. 2013) and transfected into HT22 by jetPRIME reagents (Polyplus-transfection^®^, Illkirch-Graffenstaden, France). Single clones were picked and screened for genetic and functional deletion of HT22. GRPR deleted cell clones were named as HT22-GRPR KD cells.

### Statistical Analysis

Data were presented as means and standard deviations (SDs) or medians and quantiles depending on the distribution of data. The confocal images and protein blots shown were representative data from at least three independent experiments. The values and graphs of the Pearson correlation were obtained using the GraphPad Prism. A p-value < 0.05 and | r |> 0.3 was considered statistically significant and relevant.

## Results

### GRPR is Increased in the DG Area of Hippocampus of ASD Mice

**Fig. 1:**
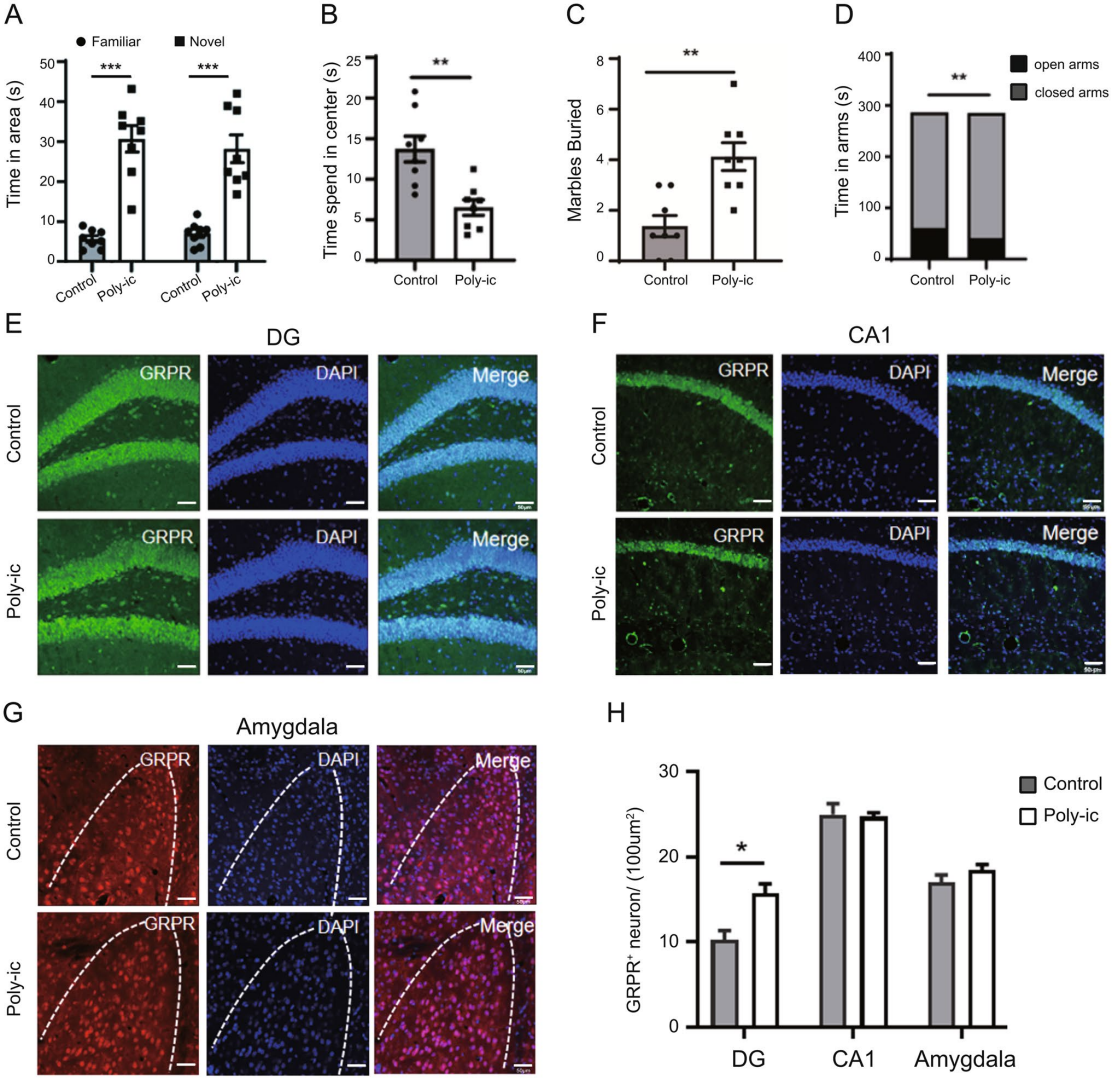
GRPR is increased in DG of the hippocampus of MIA offspring. (**A**) In the three-chamber social test, social interaction of Poly-ic group showed significantly less than control group (n = 8, *** p < 0.001). (**B**) In the open field experiment, the time of mice in the Poly-ic group staying in the central area was reduced compared with that in the control group (n = 8, ** p < 0.01). (**C**) The number of buried beads in the Poly-ic group was higher than that in the control group (n = 8, ** p < 0.01). (**D**) In the cross elevated test, the mice in the Poly-ic group spent significantly less time in the open arm area than those in the control group, while the time spent in the closed arm area increased (n = 8, ** p < 0.01). Representative images of GRPR in DG (**E**) and CA1 (**F**) of the hippocampus and amygdala (**G**). Scale bar, 50 μm. (**H**) Count the number of GRPR + neurons in each group of three independent visual fields (100µm 2) (Data are mean ± SEM, n = 6, * p < 0.05)

In this study, we examined the expression of GRPR in different brain regions of mice in the MIA model. The ASD mice from the Poly-ic group exhibited several autism-like behaviors. They showed decreased social preference in the three-chamber social interaction test (Fig. 1A), spent less time in the center area of the open field (Fig. 1B), displayed increased repetitive behaviors as observed in marble burying (Fig. 1C), and spent less time in the open arms and more time in the closed arms of the elevated plus maze (Fig. 1D). These behavioral changes indicated that the mice from the Poly-ic group exhibited autism-like behavior. Considering the connection between GRPR and ASD-like abnormal behaviors, we further investigated the expression of GRPR in the brain using immunofluorescence. Our results showed a significant increase in the expression of GRPR in the DG region of the hippocampus in the Poly-ic mice compared to the control group (Fig. 1E and H). However, there were no significant changes observed in the CA1 region of the hippocampus (Fig. 1F and H) or the amygdala (Fig. 1G and H). In conclusion, our findings suggest that the increase of GRPR specifically occurs in the DG region of the hippocampus in ASD mice.

### The Increase of GRPR is Related to the Activation of PI3K-AKT Pathway in ASD Mice

**Fig. 2:**
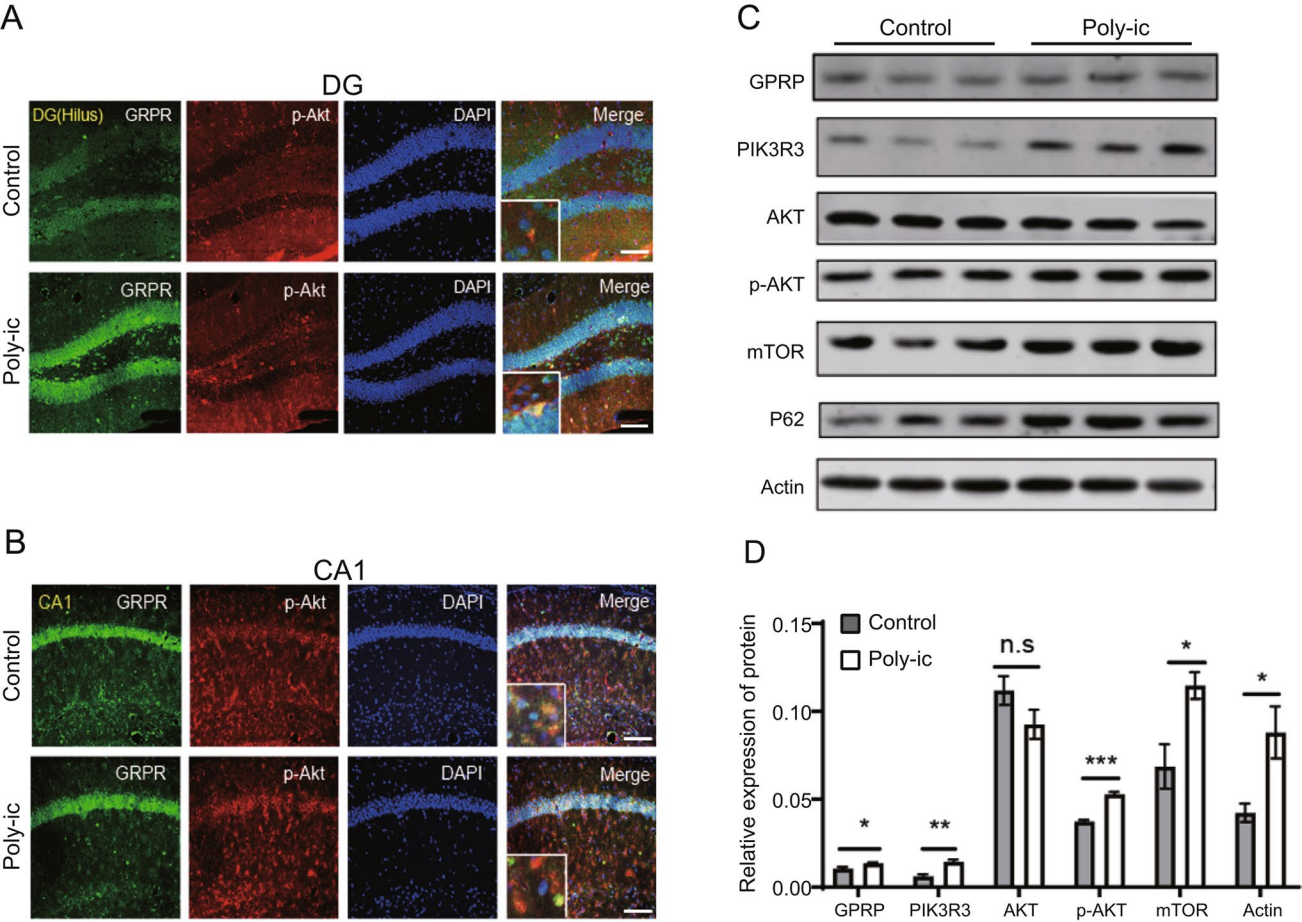
The increase of GRPR is related to the activation of PI3K-AKT pathway in ASD mice. Immunostaining shows the expression of GRPR and p-AKT in the control group and the Poly-ic group mice were co-localized (green represents GRPR, red represents p-AKT, blue represents nucleus) in DG (**A**) and CA1 (**B**) of the hippocampus of ASD mice. Scale bar = 100 μm. (**C**) Representative images showed the expressions of GRPR, PIK3R3, AKT, p-AKT, mTOR in hippocampus of control and ASD mice, and (**D**) quantified by ImageJ. Data are mean ± SEM ( n = 3, * p < 0.05, ** p < 0.01, *** p < 0.001)

To investigate the relationship between GRPR and the PI3K-AKT/mTOR signaling pathway, we examined the expression of p-AKT in the DG (Fig. 2A) and CA1 (Fig. 2B) regions of the hippocampus in mice with ASD. The results depicted in Fig. 2A and B demonstrate an increase in p-AKT levels in the hippocampus of ASD mice, which co-localized with GRPR expression. Furthermore, the protein expression of PIK3R3 and p-AKT in the hippocampus of ASD mice from the Poly-ic group was significantly higher compared to the control group. However, the expression of AKT did not exhibit any statistical change (Fig. 2C and D).

### IL-6 Increases GRPR and Activates PI3K-AKT/mTOR Pathway In vitro

**Fig. 3:**
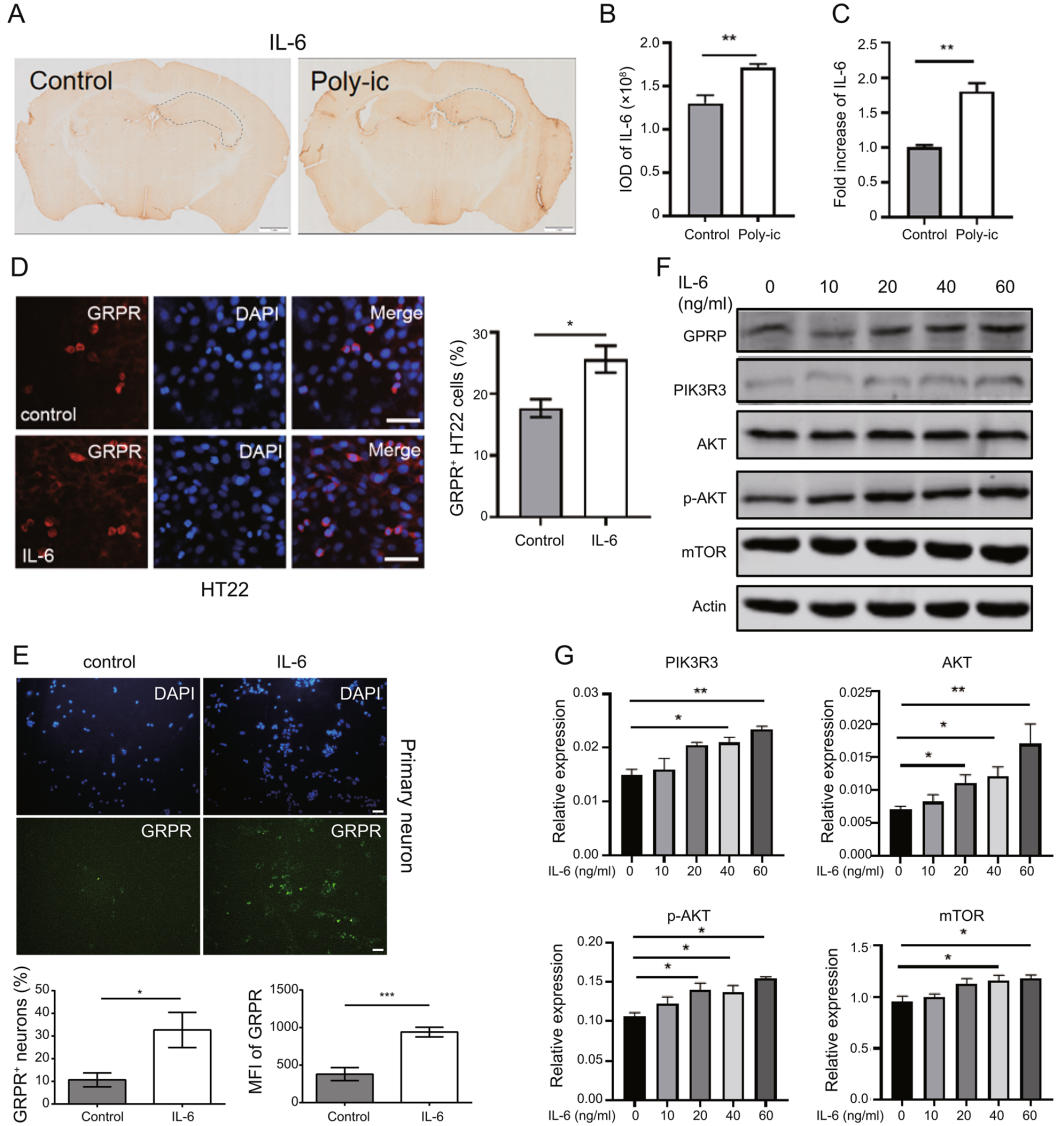
IL-6 increases GRPR and activates PI3K-AKT/mTOR pathway in vitro. (**A**) Immunohistochemistry shows that there is a difference in the expression of IL-6 in the coronal section of the brain between the control group mice and the Poly-ic group mice (the scale is 1 mm). (**B**) The average unit area optical density of IL-6 in the brain of mice in Poly-ic group was significantly higher than that in the control group ( n = 3). (**C**) Compared with the control group, the expression of IL-6 in the brain tissue of mice in Poly-ic group increased significantly ( n = 3, ** p < 0.01). After stimulated with IL-6 at a concentration of 60 ng/ml for 24 h, GRPR positive neurons of HT22 cells (**D**) and primary neurons (**E**) were increased compared with cells without IL-6 treatment (scale 20 μm). (**F**) Representative images showed the expressions of GRPR, PIK3R3, AKT, p-AKT, mTOR in HT22 cells after treated with IL-6 at indicated concentrations and (**G**) quantified by ImageJ (* p < 0.05, ** p < 0.01)

To investigate the role of IL-6 in promoting ASD-like behavior in a mouse model of MIA (Sarieva et al. 2023), we initially examined the expression levels of IL-6 in the mouse brains. Immunohistochemical analysis revealed higher levels of IL-6 in the brains of mice from the Poly-ic group compared to the control group (Fig. 3A and B), while mRNA expression of IL-6 was similar between the two groups (Fig. 3C). Moreover, upon IL-6 stimulation of HT22 cells and primary neurons in vitro, there was a significant increase in GRPR-positive cells (Fig. 3D and E). Additionally, HT22 cells exhibited significantly higher levels of PIK3R3 and p-AKT upon IL-6 stimulation (Fig. 3F and G). These findings suggest that IL-6 can up-regulate GRPR expression in HT22 cells and activate the PI3K-AKT signaling pathway, with the observed effects showing a certain correlation with the concentration of IL-6.

### Reducing GRPR in HT22 Cells can Inhibit PI3K-AKT/mTOR Pathway Activation

**Fig. 4:**
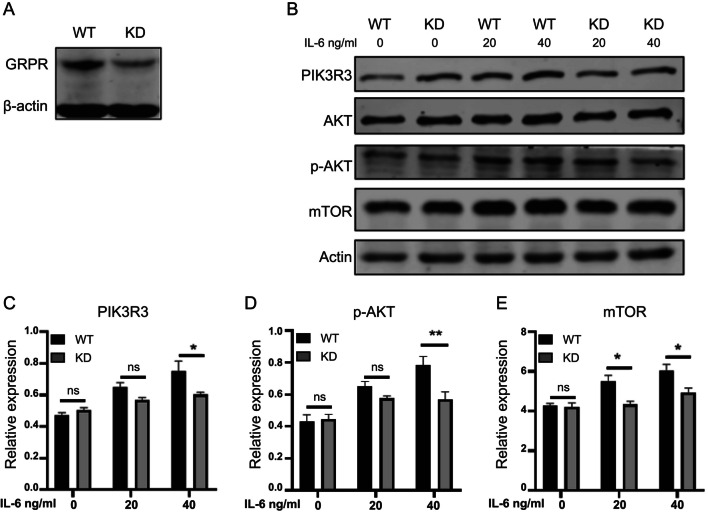
Reducing GRPR in HT22 cells can inhibit PI3K-AKT/mTOR pathway activation. (**A**) Constructing the GRPR Knockdown (KD) cells strain. (**B**) Representative images showed blots of different proteins (PIK3R3, AKT, p-AKT, mTOR) in GRPR WT and GRPR KD cells after IL-6 treatment in different concentrations (0ng/ml, 20ng/ml, 40ng/ml) for 24 h. (**C**–**E**) Relative expression of PIK3R3, p-AKT and mTOR in GRPR WT and GRPR KD cells after IL-6 stimulation in different concentrations (0ng/ml, 20ng/ml, 40ng/ml) for 24 h. Data are mean ± SEM ( n = 3, * p < 0.05, ** p < 0.01)

To investigate the role of GRPR in IL-6 activation of the PI3K-AKT/mTOR signaling pathway, we utilized CRISPR-Cas9 to knock down GRPR expression in HT22 cells. In comparison to wild-type (WT) HT22 cells, the HT22 GRPR KD cells exhibited decreased expression of GRPR (Fig. 4A). Following treatment with IL-6 (40ng/ml) for 24 h, the expression of mTOR (Fig. 4B and E) was reduced in HT22 GRPR KD cells compared to HT22 WT cells, while PIK3R3 (Fig. 4B and C) and p-AKT (Fig. 4B and D) showed no significant changes. Moreover, under low IL-6 concentration (20ng/ml, 24 h) treatment, only mTOR displayed a significant decrease in HT22 GRPR KD (Fig. 4B and E). These findings suggest that the downregulation of GRPR expression in HT22 cells can diminish the activation level of the PI3K-AKT/mTOR signaling pathway following IL-6 treatment.

### mRNA Expression of GRPR was Increased and Related to PI3K-AKT/mTOR Signaling Pathway Activation in the Hippocampus of ASD Mice

**Fig. 5:**
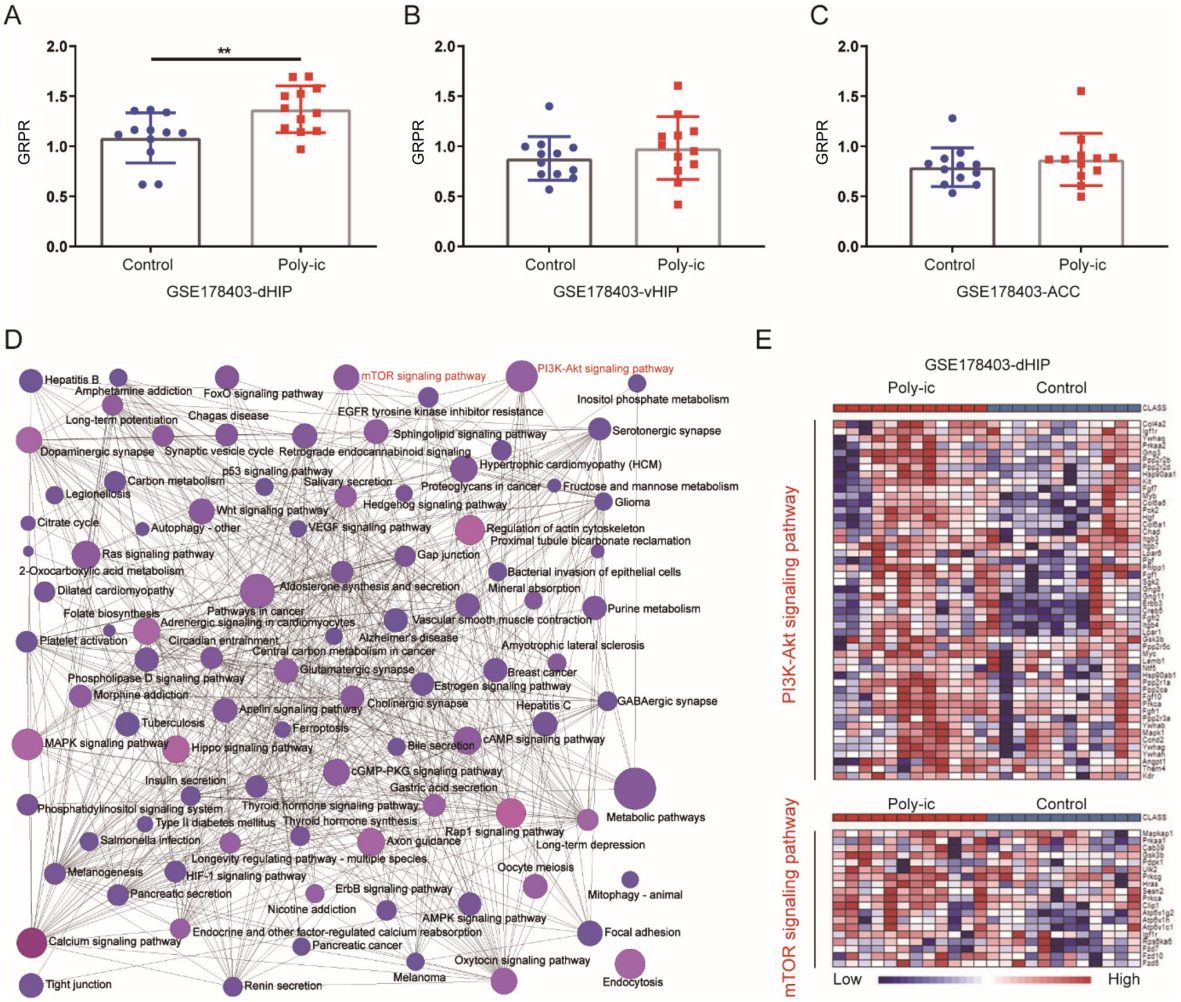
mRNA expression of GRPR was increased and related to PI3K-AKT/mTOR signaling pathway activation in the hippocampus of ASD mice. Expression of GRPR in the dorsal hippocampus (**A**), GRPR in ventral hippocampus (**B**) and anterior cingulate cortex (**C**) of samples from GSE178403. ** p < 0.01. (**D**) The enrichment network of DEGs identified using network-based analysis, the colors of nodes are positively correlated with the fold change of DEGs in the signaling pathway. (**E**) ORA-generated heatmaps of core enrichment genes in the PI3K-AKT and mTOR signaling pathway upregulated in ASD mice from the Poly-ic group of orsal hippocampus samples from GSE178403

 In our study, we examined the expression of dorsal hippocampus (dHIP) (Fig. 5A), ventral hippocampus (vHIP) (Fig. 5B), and anterior cingulate cortex (ACC) (Fig. 5C) from GSE178403. We observed a significant increase in the expression of GRPR only in the dHIP of ASD mice from the Poly-ic group. Subsequently, we used NetworkAnalyst to analyze the pathway enriched in genes positively associated with GRPR expression. Our analysis revealed that the PI3K-AKT and mTOR signaling pathways were enriched, and there was evidence of cross-reaction between these two pathways (Fig. 5D). Furthermore, we generated a heat map (Fig. 5E) to visualize the expression of genes involved in the PI3K-AKT and mTOR signaling pathway, which showed enhanced expression in mice from the Poly-ic group compared to the control group. These findings suggest that the increased expression of GRPR in the hippocampus of ASD mice may be linked to the activation of the PI3K-AKT/mTOR signaling pathway.

### Gsk3b was a Hub Gene in the PI3K-AKT/mTOR Signaling Pathway Activated by IL-6-STAT3-GRPR

**Fig. 6:**
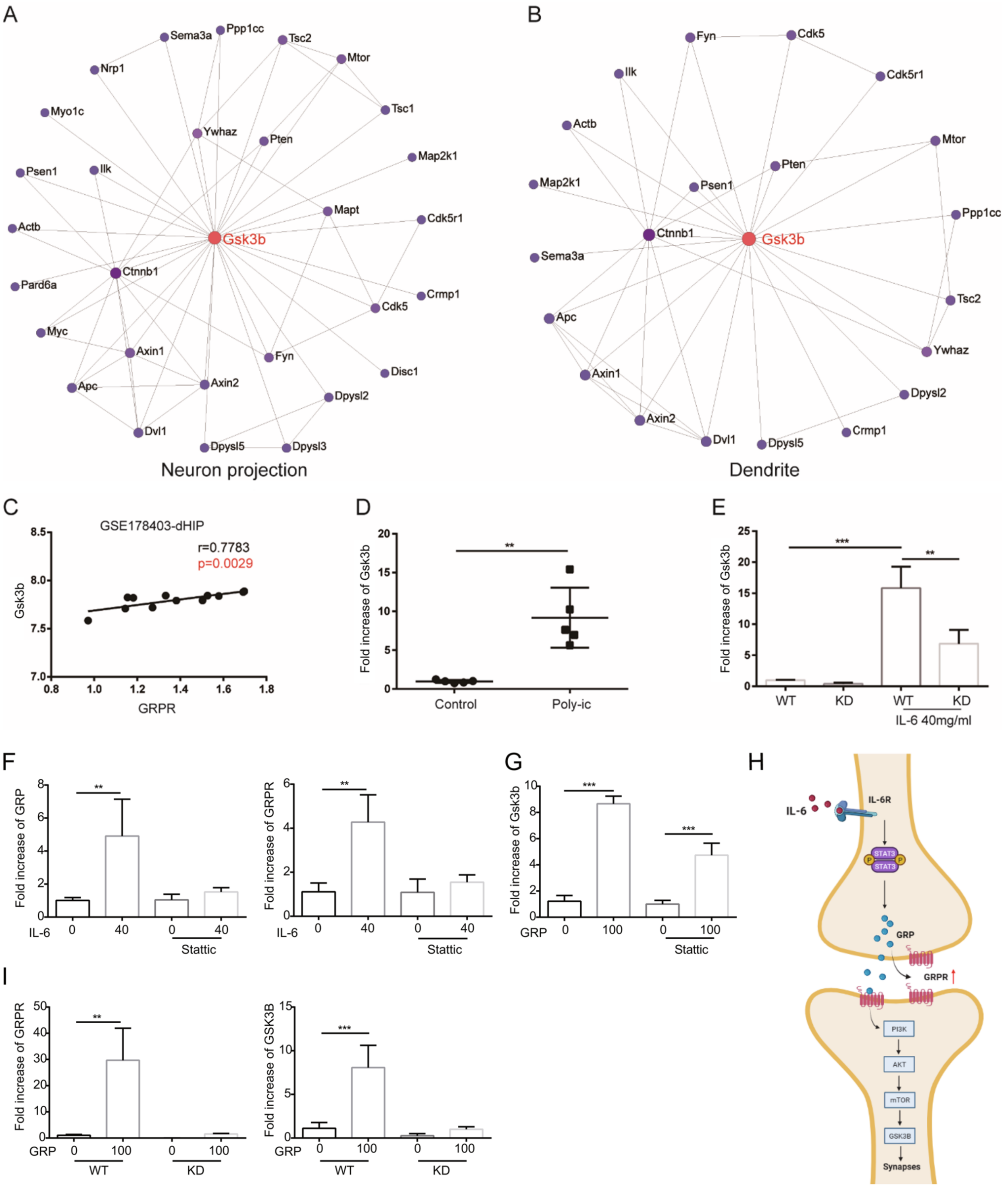
Gsk3b was a hub gene in the PI3K-AKT/mTOR signaling pathway activated by IL-6-STAT3-GRPR. Zero-order interaction network of genes involved in neuron projection (**A**) and dendrite (**B**) identified by network-based analysis. (**C**) The gene expression correlation between GRPR and Gsk3b. Pearson correlation coefficients (r) and p values were calculated and shown. (**D**) qPCR for Gsk3b in the entire hippocampus of MIA male offspring ( n = 5). (**E**) qPCR for Gsk3b in the HT22 and HT22-GRPR KD with or without IL-6 treatment ( n = 5). (**F**) After treated with or without Stattic (STAT3 inhibitor, 10 µM, 4 h), HT22 cells were stimulated by IL-6 (40 ng/ml, 24 h) to detect the mRNA expression of GRP and GRPR. (**G**) After treated with or without Stattic (STAT3 inhibitor, 10 µM, 4 h), HT22 cells were stimulated by recombinant mouse GRP (100 nM, 24 h) to detect the mRNA expression of Gsk3b. (**H**) Schematic diagram of IL-6 promoting GRP-GRPR and Gsk3b through STAT3. (I) mRNA expression of GRPR and Gsk3b in HT22-WT and HT22-GRPR KO cells after GRP (100 nM, 24 h) treatment. Representative data from three independent experiments were shown (* p < 0.05, ** p < 0.01, *** p < 0.01)

To investigate the role of GRPR-mediated PI3K-AKT/mTOR activation in ASD, we selected genes that showed a positive correlation with GPRP expression in the AKT pathway. We then performed GO analysis and found that two pathways, neuron projection and dendrite, were enriched in these genes. Subsequently, we analyzed the genes involved in the neuron projection and dendrite pathways using NetworkAnalyst. As depicted in Fig. 6A and B, glycogen synthase kinase 3 β (Gsk3b) emerged as the top-ranked gene in both networks based on the Degree of centrality and betweenness (Li et al. 2022). It has been well-established that activated AKT can interact with numerous downstream proteins, and GSK-3β has been confirmed to play a role in neuronal apoptosis signaling (Razani et al. 2021). Furthermore, Fig. 6C demonstrates a significant positive correlation between Gsk3b and GRPR in the dHIP from GSE178403. We also observed a significant increase in the mRNA expression of Gsk3b in the hippocampus of ASD mice (Fig. 6D). Additionally, upon IL-6 stimulation in vitro, the expression of Gsk3b was significantly reduced in HT22-GRPR KD compared to HT22 (Fig. 6E).

To further investigate the mechanism of IL-6-mediated GRPR activation in ASD, we applied Stattic, a phosphorylation inhibitor of IL-6 downstream signaling transcription activator STAT3. We observed that Stattic can inhibit the increased mRNA expression of GRP and GRPR in HT22 cells under IL-6 stimulation (Fig. 6F). However, IL-6 still activates the expression of Gsk3b in HT22 cells, regardless of the presence of Stattic (Fig. 6G). These findings suggest that IL-6 may increase the expression of GRPR in neurons by upregulating their expression of GRP (Fig. 6H). Additionally, to assess the impact of GRP on neurons, we stimulated HT22 cells with recombinant mouse GRP. We observed that GRP can enhance the expression of GRPR and Gsk3b in HT22 cells, but not in HT22-GRPR KO cells (Fig. 6I).

## Discussion

Our results demonstrate that increased expression of GRPR leads to the activation of the PI3K-AKT/mTOR-GSK-3β signaling pathway in the hippocampus of ASD mice. Additionally, our findings indicate that IL-6 promotes PI3K activation by upregulating GRP/GRPR expression. Therefore, our study establishes a connection between elevated IL-6 levels in the hippocampus, upregulated GRPR, and overactivated PI3K-AKT/mTOR-GSK-3β signaling in ASD mice from MIA. This suggests a potential role of GRPR in promoting ASD-like behavioral abnormalities.

Accumulating evidence suggests a strong association between IL-6 and ASD, although the precise mechanism of IL-6 on neurons in specific brain regions of ASD remains unclear (García-Juárez and Camacho-Morales 2022). Frozen brain tissue in individuals with ASD has shown significantly elevated levels of IL-6 compared to healthy controls (Li et al. 2009), and the secretion of IL-6 has been found to be positively correlated with the severity of ASD (Hu et al. 2018). IL-6 can increase the number of excitatory synapses by promoting mature dendritic spines, leading to an imbalance in excitation-inhibition in synaptic transmission (Hu et al. 2018). Furthermore, IL-6 has been identified to play an important role in ASD offspring of mothers with MIA (Sarieva et al. 2023). MIA leads to increased secretion of the pro-inflammatory cytokine IL-6 in the serum, which can further increase IL-6 levels in the fetus through the placental barrier (Wu et al. 2017). Enhanced IL-6 and STAT3 phosphorylation have been observed in the fetal brain of MIA mice, and knocking out the IL-6 receptor has been shown to reduce marble-burying repetitive behavior and rescue social exploration reductions caused by MIA (Wu et al. 2017). Our experiment found a significant increase in IL-6 levels in the hippocampus of ASD mice (Fig. 3), and IL-6 was shown to increase the expression of GRP-GRPR and PI3K-AKT/mTOR-GSK-3β in HT22 cells (Fig. 6). Therefore, this study provides an important clue to the neural mechanism by which MIA causes ASD-like behavioral abnormalities through IL-6.

As one of the potential pathogenic genes of autism, GRPR has received increasing attention in the field of neurological research (Presti-Torres et al. 2012). Studies on synaptic excitatory-inhibitory imbalance have found that GRPR, located in the lateral amygdala nucleus, is mainly expressed in GABA interneurons. This expression can improve the excitatory-inhibitory imbalance of neurons by exciting such neurons and then inhibiting their downstream neurons (Shumyatsky et al. 2002; Roesler and Schwartsmann 2012). Stimulation with continuous GRPR antagonist (RC-3095) in neonatal rats 10 days before birth can lead to major autistic symptoms in adulthood, such as decreased social skills, stereotypical behavior, and increased learned fear memory (Presti-Torres et al. 2012). GRPR in the dorsal hippocampus impacts the process of memory through PKC, MAPK, and PKA-related signaling pathways (Roesler et al. 2006a, b). In our experiments, we observed an increase in GRPR expression in the hippocampus of MIA offspring and dHIP from GSE178403(Figs. 1 and 5). We also demonstrated that IL-6 upregulates the expression of GRP/GRPR in neurons through the STAT3 pathway (Figs. 3 and 6). Therefore, the enhanced expression of GRPR in hippocampal neurons may be an important mechanism by which MIA leads to abnormal ASD behavior through IL-6.

PI3K plays a crucial role in mediating GRPR function (Sun et al. 2023). It is involved in synaptic plasticity and memory formation in various brain areas, including hippocampal Schaffer-CA1 synapses, dentate gyrus, and amygdala (Chen et al. 2005). PI3K is responsible for important events in memory formation, such as the insertion of AMPA receptors into the postsynaptic membrane and the initiation of protein synthesis (Chen et al. 2005; Sánchez-Castillo et al. 2022). Activation of the PI3K pathway promotes cell survival, acts on downstream mTOR to facilitate protein synthesis, and may be implicated in synaptic plasticity and memory consolidation (Horwood et al. 2006). Injecting PI3K inhibitors into the hippocampus of rats has been shown to impede the extraction and regression of contextual conditioning fear memories, indicating the significance of PI3K activation in the maintenance of this type of memory (Chen et al. 2005; Kritman and Maroun 2013). Our study discovered that GRPR and p-AKT were co-localized in the hippocampus of MIA offspring and were enhanced by IL-6 in HT22 (Figs. 2 and 3). By reducing the expression of GRPR in HT22, we observed inhibition of the activation of the PI3K-AKT/mTOR signaling pathway (Fig. 4). These findings suggest that IL-6 enhances GRPR in hippocampal neurons of MIA offspring mice, potentially regulating neural function through the PI3K-AKT/mTOR signaling pathway.

GSK-3β is a crucial kinase regulated by the PI3K-AKT signaling pathway, which has significant effects on synapses and cognitive function (Cao et al. 2019). The PI3K/AKT/GSK-3β signaling pathway has emerged as a key regulatory factor in dendritic spinal dynamics and neuron projection (Fang et al. 2011; Swiatkowski et al. 2017). Suppression of AKT/GSK-3β signal transduction has been found to result in the loss of dopaminergic neurons (Zhu et al. 2019). Recent research indicates that overactivation of GSK-3β could impair the developing hippocampus and contribute to cognitive deficits (Abbah et al. 2022). Increased GSK-3β in GABAergic interneurons has been associated with abnormal spatial working memory (Nakao et al. 2020). Activation of the PI3K-AKT signaling pathway leads to an increase in phosphorylated GSK-3β (Jaworski et al. 2019). Recent study demonstrated that activation of the PI3K-AKT signaling pathway in ASD promotes an increase in phosphorylated GSK-3β, which regulates neuronal survival and differentiation (Ahmed et al. 2023). Interestingly, our results show that in the hippocampus of ASD mice with increased GRPR expression, both neuron projection and dendrite pathways are enriched, with GSK-3β being the critical regulatory molecule (Figs. 5 and 6). While we have demonstrated that the activation of GRPR in HT22, promoting the mRNA expression of GSK-3β (Fig. 6), we have not yet determined whether phosphorylated GSK-3β increases or how GSK-3β affects the survival and synaptic formation of hippocampal neurons in ASD mice. Therefore, it is worth investigating the association between GSK-3β signaling in hippocampal neurons and abnormal behavior in ASD mice, a s well as developing targeted interventions to alleviate MIA-induced ASD.

 In summary, our study demonstrates that IL-6 can enhance the activation of the PI3K-AKT/mTOR-GSK-3β pathway in hippocampal neurons of ASD mice by upregulating GRPR. Thus, GRPR may affect the development of ASD through the regulation of GSK-3β in neurons within the inflamed brain. Our findings suggest that GRPR could serve as an important target for future research.

Schematic diagram of the signaling pathways involved in IL-6 activating the PI3K-AKT/mTOR-GSK-3β pathway in hippocampal neurons of ASD mice. Increased IL-6 in the hippocampus of MIA offspring mice leads to increased GRP-GRPR signaling in neurons, which in turn stimulates the activation of the PI3K-AKT/mTOR-GSK-3β signaling pathway. This activation may affect signaling in neuron projection and dendrite development.

## Data Availability

The datasets used and/or analyzed during the current study are available from the corresponding author on reasonable request.
